# Vaccinal Syphilis

**Published:** 1873-11-01

**Authors:** Lyman Ware

**Affiliations:** Chicago


### VACCINAL SYPHILIS.

Editors Examiner: — Knowing full well that there is no greater error, nor none more mischievous, than drawing conclusions from imperfect or meager data, yet, on account of the recent discussions on vaccinal syphilis, and as it may be some time before a similar opportunity may present itself, I venture to give my observations, however limited they may be.

In the early part of December, 1871, during the epidemic of small pox, Mr. A—— began treatment for secondary syphilis; and as he had no knowledge of ever having been vaccinated, bovine virus was inserted about the same time, and a beautifully marked pustule resulted, which was allowed to harden into a scab. When the scab became detached the exterior was carefully scraped, that there might be no admixture of blood or extravenous matter. As I had several years previously had a severe attack of varioloid, I did not expect the vaccine virus would have any effect upon me; but if syphilis could be transmitted by the virus, no shadow of a cause existed to prevent it.

A portion of the scab was carefully dissolved and inserted in the left forearm. Aside from a slight redness which occurred a few days subsequently, nothing ever resulted.

The scab was placed in glycerine, and after an elapse of ten weeks, as not the slightest symptom was manifested in me, six infants at the dispensary were vaccinated with it and kept under observation. Of the six, four exhibited well formed pustules, and the remaining two were vaccinated with other virus. After a period of three months none of them showed the faintest appearance of specific disease. If bovine virus comes into general use, as it now seems likely to do, the question of vaccinal syphilis will have little interest. Of the four conditions assumed by Mr. Jonathan Hutchison as necessary to the transmission of syphilis, the fourth only is wanting.

"1st. A syphilis Vaccinifer; 2d. An active condition of the syphilitic element of the vaccinifers blood, but at the same time, 3d, An absence of such external symptoms of syphilis as would deter any commonly upright surgeon from using the subject of them as a vaccinifer; 4th. The gross imprudence committed of employing either blood or the serum obtained after the emptying of the vericle."

LYMAN WARE, M.D.

Chicago, October, 1873.

